# Foot-and-Mouth Disease Virus: Immunobiology, Advances in Vaccines and Vaccination Strategies Addressing Vaccine Failures—An Indian Perspective

**DOI:** 10.3390/vaccines7030090

**Published:** 2019-08-16

**Authors:** Raj Kumar Singh, Gaurav Kumar Sharma, Sonalika Mahajan, Kuldeep Dhama, Suresh H. Basagoudanavar, Madhusudan Hosamani, B P Sreenivasa, Wanpen Chaicumpa, Vivek Kumar Gupta, Aniket Sanyal

**Affiliations:** 1 ICAR-Indian Veterinary Research Institute, Izatnagar, Uttar Pradesh 243122, India; 2 ICAR-IVRI Bangalore Campus, Bangalore, Karnataka 560024, India; 3 Center of Research Excellence on Therapeutic Proteins and Antibody Engineering, Department of Parasitology, Faculty of Medicine Siriraj Hospital, Mahidol University, Bangkok 10700, Thailand

**Keywords:** foot-and-mouth disease, immunobiology, vaccines, vaccination failure, virus emergence

## Abstract

A mass vaccination campaign in India seeks to control and eventually eradicate foot-and-mouth disease (FMD). Biosanitary measures along with FMD monitoring are being conducted along with vaccination. The implementation of the FMD control program has drastically reduced the incidence of FMD. However, cases are still reported, even in regions where vaccination is carried out regularly. Control of FMD outbreaks is difficult when the virus remains in circulation in the vaccinated population. Various FMD risk factors have been identified that are responsible for FMD in vaccinated areas. The factors are discussed along with strategies to address these challenges. The current chemically inactivated trivalent vaccine formulation containing strains of serotype O, A, and Asia 1 has limitations including thermolability and induction of only short-term immunity. Advantages and disadvantages of several new-generation alternate vaccine formulations are discussed. It is unfeasible to study every incidence of FMD in vaccinated animals/areas in such a big country as India with its huge livestock population. However, at the same time, it is absolutely necessary to identify the precise reason for vaccination failure. Failure to vaccinate is one reason for the occurrence of FMD in vaccinated areas. FMD epidemiology, emerging and re-emerging virus strains, and serological status over the past 10 years are discussed to understand the impact of vaccination and incidences of vaccination failure in India. Other factors that are important in vaccination failure that we discuss include disrupted herd immunity, health status of animals, FMD carrier status, and FMD prevalence in other species. Recommendations to boost the search of alternate vaccine formulation, strengthen the veterinary infrastructure, bolster the real-time monitoring of FMD, as well as a detailed investigation and documentation of every case of vaccination failure are provided with the goal of refining the control program.

## 1. Introduction

Foot-and-mouth disease (FMD) is a highly contagious and economically important disease of cloven-hoofed domestic and wild ruminants including cattle, buffalo, swine, goats, and sheep [1,2]. High body temperature and appearance of vesicular lesions on oro-nasal mucosa, interdigital cleft, coronary band, udder, and teats epithelium are primary characteristics of the disease. FMD results in loss of appetite and body weight, milk yield, draft power, and abortions in advanced pregnant animals [3]. Indirect losses include trade loss due to ban on export of milk and milk products and other animal products into FMD-free countries. Morbidity reaches 100% in herds that have not been vaccinated. Mortality is significantly high (>20%) in young calves due to myocarditis [4,5]. Often, a substantial proportion of recovered animals become carriers of FMD virus (FMDV) [6,7,8].

**Figure 1:**
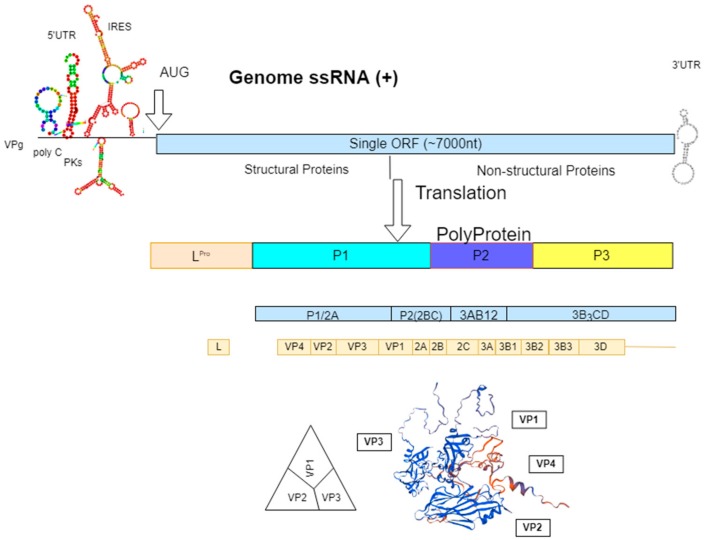
Schematic drawing showing the genome organization and structure of foot-and-mouth disease virus (FMDV), displaying structural and non-structural proteins. Nucleotide sequence of virus serotype O (IND228/14) was used for RNA secondary structure folding using RNAfold webserver (http://rna.tbi.univie.ac.at) and structural protein refolding using Swiss Model (https://swissmodel.expasy.org).

**Figure 2:**
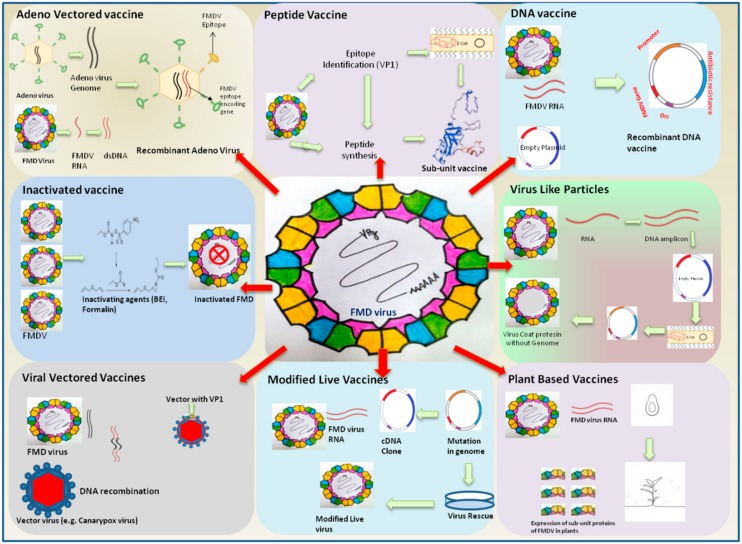
Trends in advances in vaccines against FMDV. Inactivated vaccines are most widely used, but, as they suffer from several demerits, the search for new generation vaccines is on. Several alternatives such as Adeno virus vectored vaccine and virus-like particles have been shown to be promising considerationsfor FMD control in India. The brief procedure used for development of these vaccines is depicted in the figure.

The causative agent is FMDV, which belongs to the genus *Aphthovirus* in the family *Picornaviridae*. The virus occurs as seven genetically and antigenically distinct serotypes—O, A, C, Asia 1, and Southern African Territories (SAT) 1–3. Each serotype has multiple subtypes within each serotype [2]. The viral genome is approximately 8.3 kb in length and is enclosed within a protein capsid. The RNA genome contains a large open reading frame that encodes four viral structural proteins (VP1, VP2, VP3, and VP4) from the P1 polypeptide and seven non-structural proteins (L_pro_, 2A, 2B, 2C, 3A, 3b, 3C_pro_, and 3D_pol_) from the P2 and the P3 polypeptides (Figure 1; [9]). The 5′ and the 3′ untranslated regions (UTRs) are important for viral replication and translation [10,11,12]. The capsid contains 60 copies each of four different structural proteins (VP1–4). VP1–3 are surface exposed, while VP4 is internalized (Figure 1; [2]). The crystallographic structure of the FMDV capsid revealed that immunological epitopes are mostly found on surface-oriented interconnecting loops between structural elements [13]. The highly conserved Arg-Gly-Asp (RGD) amino acid motif within the G-H loop plays a major role in viral entry into host cells [10] and contributes to protective immunity in the host [14,15]. The αV family of integrin receptors binds with the G-H loop for receptor-mediated viral entry. Absence of this receptor on host cells diminishes the ability of viral entry [16,17]. The low fidelity RNA polymerase leads to error prone viral replication and genome mutations. The involvement of immunologically critical sites can lead to the emergence of immunological variants of FMDV. Immunity against one serotype does not provide protection against other serotypes or sometimes to the variants within the same serotype [15].

Control and eventual eradication of FMD is essential for sustainable farm and economic growth. Broad host range, epidemiological complexity, increasing movement of animals and animal products in the international market, high infectivity rate, emergence of new variants, interface of domestic and wild animals, lack of highly potent vaccines, and lack of required infrastructure are some of the key factors that hinder the control and the eradication of FMD in endemic settings [18]. Compulsory vaccination with strict sanitary measures has been successfully implemented for the control or the eradication of FMD in Europe and South American countries [18,19]. Depopulation of FMD infected and in-contact animals is preferred over introduction of vaccination in countries that are free of FMD [20]. However, more recently, a “vaccinate to live” policy has also been considered in countries that are free from FMD due to social issues involved in large-scale culls [20]. In a unique case in the Netherlands, the “vaccinate to kill” policy was adopted, where in-contact animals were vaccinated followed by killing when the outbreaks subsided. The action produced quick control over the complete “test and kill” policy [21]. However, policies intended to control FMD in endemic settings featuring the simultaneous circulation of more than one serotype should differ from the situation where one FMDV serotype is introduced in FMD-free areas.

FMD is endemic in India. The oldest documented report of FMD in the country dates back to 1864 [22]. The direct estimated economic losses due to FMD are USD 2.0–3.2 billion per annum [23,24,25]. Indirect losses due to trade barriers will further escalate this figure. FMD in India is a complex issue, and several challenges need to be tackled to control and eradicate the disease from the country. Prevalence of multiple serotypes with constant emergence of new variants, lack of highly potent and stable vaccines, large population, species interface, and lack of required number of trained personnel are some of these challenges. In addition, the test and kill policy is not being considered in India due to socio-economic issues [26]. The World Organization for Animal Health and Food and Agriculture Organization of the United Nations (OIE/FAO) recommends the progressive control pathway (PCP), a staged approach for the control of FMD in endemic settings. FMD-PCP consists of stages I to V for successful reduction, elimination, and eradication of the disease [27]. India is currently in stage III of the PCP with control of virus circulation by active immunization [26].

India has been striving to control FMD since 2003, when a systematic, mass vaccination-based FMD control program (FMDCP) was launched as a pilot project in 54 districts [26,28]. Subsequently, by 2017–2018, the program was expanded to cover the entire country. The number of occurrences of the disease and the intensity of infection, measured in terms of number of animals affected and recovery time, has declined with the implementation of the FMDCP [29]. However, FMD is still prevalent with variable intensities in almost all parts of the country, including areas covered under FMDCP [30,31]. Despite repeated vaccinations at 4–6 month intervals, outbreaks of FMD persist in the vaccinated herds [30,32]. Various factors are associated with the incidence of FMD in the areas covered by vaccination [33,34].

The current, chemically inactivated trivalent vaccine against serotype O, A, and Asia 1 suffers limitations, including the requirement of a biosafety level III facility for mass production of virus antigen, thermolability, and only short-lived immunity. Ongoing efforts worldwide—including in India—are trying to refine the existing vaccine or to develop an alternate vaccine formulation for effective vaccination. Some of the recent approaches, including virus-like particles, gene-deleted modified virus vaccine, and vector-mediated vaccines, have been explored with variable success. Other options that have been advocated include the use of vaccines without handling live virus, such as DNA vaccine, peptide vaccines, and sub-unit vaccines. Presently, all these vaccines are in the experimental stage and have not been tested in field.

The present review describes the current advances and the immunobiology of FMDV vaccines, vaccination strategies, factors responsible for FMD vaccination failures, and strategies to mitigate FMD vaccination challenges. The details of both conventional and new-generation advances in FMDV vaccine design and formulation are discussed from the perspective of the use in India. The impacts of vaccination on FMD epidemiology and sero-epidemiology are also highlighted. Various factors for vaccination failure are detailed. Understanding the process of the emergence of new variants during the vaccination campaign is essential, and we focus on the three prevalent serotypes in India. Other factors including strengthening veterinary infrastructure in the country, policies related to the use of monovalent vaccines, and vaccines targeting multiple diseases, including hemorrhagic septicemia, are discussed. It is crucially important to understand the advantages and the disadvantages of new-generation and conventional FMD vaccines, the details of the epidemiology of FMD in India, and the limiting factors accounting for vaccine failures and design of strategies to counter failures and strengthen the available infrastructure in India to implement an effective FMD prevention and control program.

## 2. Trends and Advances in Vaccines against FMDV

The most important factors in a vaccination-based control program are the type and the quality of the available vaccines. FMD vaccine was one of the first animal vaccines to be developed in the early 19th century and is one of the most widely used of all mammalian vaccines, with over two billion doses produced annually [35]. The available vaccines do not prevent primary infection and offer protection only from generalized clinical disease. Exposure of FMDV in the vaccinated animals results in infection without the appearance of clinical symptoms, and often animals acquire the carrier stage where it sheds virus silently [36,37]. However, a highly potent and safe vaccine is still awaited [38]. Advances in FMD vaccines have been discussed from time to time [39,40,41,42]. In this section, we briefly review the various conventional and new-generation vaccines available and assess their merits, disadvantages, and feasibility for use for FMD control in India (Figure 2). Currently, whole virus inactivated vaccine is used in the FMDCP in India. The search for an alternative vaccine or refinement of the existing vaccines suiting Indian conditions is ongoing. Advancements in vaccinology have paved way to designing and developing effective FMD vaccines by new approaches such as DNA vaccines, plant based edible vaccines, vectored vaccines, subunit vaccines, virus-like particles vaccines, peptide vaccines, and others [19,40,43,44,45,46,47]. However, their successful application as alternatives to the whole virus based conventional vaccine is still to be demonstrated.

### 2.1. Inactivated Whole Virus Vaccines

Inactivated FMD vaccines are the most commonly used globally. Typically, the whole virus is grown in suspension culture and inactivated chemically, mostly by binary ethylenimine (BEI) and mixed with adjuvants [41]. Water-, oil-, and aluminum-based formulations are available. Most often, more than one serotype is included in the vaccine formulation depending upon the manufacturer and the epidemiological situation in the country. Initially, a polyvalent aqueous aluminum hydroxide and saponin adjuvant quadrivalent vaccine comprising serotypes O, A22, C, and Asia 1 was utilized in India. Quadrivalent vaccines consisting of serotypes of O, A, C, and Asia 1 were produced until 2003. Currently, a vaccine formulation containing three strains of serotypes O, A, and Asia 1 is used in India. More detailed information about specific serotype combinations used in the different regions of the world is reviewed elsewhere [20]. The antigen payload in vaccine formulations varies from three to six PD_50_. Extremely concentrated vaccines with 6PD_50_ that provide protection within one week of administration are helpful in case of outbreaks in FMD free areas. Stringent quality control is equally important for vaccine strain selection to ensure adequate protection. The FAO and the OIE recommended guidelines for quality control testing include identity, sterility, safety, potency, efficacy, and detection of FMDV non-structural proteins. The disadvantages associated with use of inactivated vaccine formulation include the short duration of immunity, thermolability, and the need for a highly regulated biosafety level III facility to prevent virus leakage during vaccine production [36]. In addition, even purified vaccine formulation sometimes may contain traces of non-structural proteins (NSPs), leading to induction of antibodies against NSPs and interference with the differentiation of infected from vaccinated animals(DIVA) assay [48,49,50,51].

### 2.2. Modified Virus Inactivated Vaccines

Various groups have developed inactivated vaccines with a modified virus to overcome the limitations of wild type virus inactivated vaccines, such as the lack of foolproof DIVA and the need for a biosafety level III facility [52]. These vaccines provide DIVA compatibility without the need to purify non-structural proteins [53]. In addition, the virus can be grown in a cell culture system but is harmless to the target animals, which lessens the need for a biosafety level III facility for vaccine manufacture [19]. With the increased knowledge of viral virulence factors, new modified viruses in the form of attenuated vaccines are being developed [21]. Initially, a modified virus lacking the leader protease coding region (leaderless) followed by BEI inactivation was shown to provide immunity that was equal to that of the wild type virus [54,55]. Subsequently, Uddowla and coworkers [56] further modified the leaderless virus by mutating the 3B and the 3D proteins to improve DIVA capability. On the similar lines, deletions of some residues in the 3A protein demonstrated equal effectiveness in pigs but still needs to be tested in cattle [57].

Similar studies have been initiated in India during the past few years with the aim of exploiting the existing vaccines used in the control program. In one study, large portions of the 3A and the 3B proteins were deleted from the Indian vaccine strain of serotype O (IND/O/R2/1975), and the comparable growth kinetics of the deleted and the wild type versions were demonstrated in cell culture [58]. Similarly, a portion of 3A at the C-terminal region and complete 3B1 and 3B2 were deleted from the serotype Asia 1 vaccine strain (IND/Asia 1/491/1997). The mutant virus demonstrated comparable infectivity titer to that of the wild type virus in the cell culture system [52]. The deleted portions of 3A and 3B were utilized to detect antibodies as a companion DIVA assay in both studies. In another study, a 6x histidine-tag coding sequence was inserted in the VP1 G-H loop of an vaccine strain of serotype O (IND/O/R2/1975) [58]. The modification allowed rapid purification of the virus using immobilized metal affinity chromatography techniques with significantly reduced DNA and host protein contaminations [58]. These mutant viruses need to be tested in animals as vaccine candidates before their application in vaccine formulations. Though such deletion markers vaccines offer better DIVA compliance, they still need biosafety handling for mass production.

### 2.3. Live Attenuated Vaccines

The biggest advantage of any live attenuated vaccines (LAVs) is their ability to induce long-term immunity. Initial LAVs were high cell culture passage viruses [59]; however, they were not further used considering the probability of reverting back to the virulent form [21]. Advancement in molecular virology made it possible to alter the genes responsible for virulence, reducing chances of reversion in the host [19]. Workers have created modified viruses by mutating deleterious genes, altering replication fidelity, and deoptimizing codons, and these demonstrated protection in animals with variable degrees of success [60]. Modification in the leader protein of FMDV or by changing one of the two translation initiation sites in the virus has been demonstrated, though studies in animals are limited [56,60,61,62,63,64]. It was demonstrated that RNAs carrying the deletion of the stem loop in the 3′ UTR on a serotype-O FMDV genome were innocuous when inoculated in pigs but elicited specific humoral and cellular immune responses [65].

Attenuation of polio virus was demonstrated by codon deoptimization [66,67]. This new approach offers higher safety of the FMD vaccine strains without affecting the antigenicity [68] in addition to containing a marker sequence to distinguish the vaccine strain from field isolates. In spite of initial success, there remains much to do before a successful live attenuated vaccine is developed against FMD.

### 2.4. Viral Vector Vaccines

Several groups used mammalian viral vectors, including poxvirus, herpes virus, and adenoviruses, to deliver FMDV sequences for expression of structural proteins in the vaccinated animals, leading to induction of effective immune response directed to FMDV [19]. Vaccinia viruses and human adenoviruses have been the most commonly used viral vectors [69]. Only partial protection was obtained in initial studies, while subsequent modifications produced better quality vaccines [70,71,72,73]. To date, recombinant-replication defective human adenovirus is the most promising vector capable of delivering the FMDV capsid sequence into the animals. The adenovirus vector carrying capsid and 3Cpro coding region has achieved complete protection in pigs and cattle [74,75,76,77,78]. In endemic settings, booster injection in cattle can further improve the neutralizing antibody response. This is a key factor that is essential if a vaccine is to be used in endemic settings in India. The vaccine is still refined by inclusion of a full-length 2B coding region, by the improved synthesis of capsid proteins of FMDV [79,80], or by inclusion of an extra RGD motif for improved transduction of adenovirus in immune dendritic cells [81].

Efforts were made to develop an adenovirus-mediated vaccine delivery system for Indian vaccine strains of FMDV. Recombinant adenovirus type 5 expressing capsid and 3Cpro were developed for all three current Indian vaccine strains in collaboration with the United States Department of Agriculture Plum Island Animal Disease Center laboratory (USDA, PIADC). Monovalent and trivalent formulations (combined application of individual recombinant virus constructs for each of the three serotypes) of the vaccine were tested in Indian cattle. The data suggested that the monovalent recombinant Ad5-FMD vaccine showed immunogenicity on single administration, while the results of the animal trial involving the multivalent vaccine were not encouraging [82]. Recombinant adenovirus-mediated FMDV capsid delivery is one of the most promising strategies of the new-generation vaccines, and the monovalent formulation could be utilized for FMD outbreaks in areas that had formerly been FMD-free. However, its application in endemic settings is still a matter of discussion, as variable immune responses were obtained for different virus serotypes, which was associated with the variable efficiency of the polyprotein (P1-2A) [82].

### 2.5. Virus-Like Particle Vaccines

Virus-like particles (VLPs), which are also termed empty viral capsids, lack nucleic acid and are naturally produced in vitro. VLPs offer several advantages, including enhanced DIVA capability, reduced need for a biosafety level III facility, and economy. Conventionally, VLPs are produced in a baculovirus expressing system followed by purification. VLPs developed for an Indian vaccine strain protected guinea pigs against homologous challenge [83]. Several improvements have been made in the production of VLPs using the dual promoter vector, 3Cpro [84], or using a bicistronic complementary DNA cassette containing two open reading frames encoding an FMDV capsid gene (P1-2A) and 3Cpro separated by an internal ribosome entry site [85]. In another study, the mutation in the VP2 region increased the thermostability of VLPs and produced sufficient protection in guinea pigs [86]. Rabbit hemorrhagic disease virus has been successfully shown to be a good platform for expression of VLPs for inducing immune responses against an inserted foreign epitope in mice. A study demonstrated good immune response using chimeric VLPs containing T cell epitope of the 3A protein of FMDV [87]. However, its feasibility and application for large scale production and use in endemic settings needs to be investigated.

Other studies used recombinant silkworm baculovirus containing the intact P1-2A and3C protease coding regions of FMDV serotype Asia 1 or serotype A to immunize and successfully protect cattle [88]. VLPs of Indian vaccine strains have also been expressed in Eri silkworm but have not been tested in animals [89]. As with adenovirus vector-mediated delivery, VLPs have potential value as an alternative to conventional inactivated vaccines. The greatest advantage is the cost savings over other alternatives. Recently, Xiao et al. [90] demonstrated the utility of VLPs expressed in a prokaryotic system to protect cattle.

### 2.6. DNA Vaccines

Theoretically, DNA vaccines offer several advantages, including rapid incorporation of gene sequences of more than one virus strain/serotype, enhanced thermostability, incorporation of marker genes, and, most importantly, the freedom from the need for a biosafety level III facility for production. However, several challenges need to be addressed before adopting the vaccine for field use [43]. Early studies using DNA encoding the entire capsid along with 3Cpro demonstrated that a large amount of DNA along with multiple inoculations are required to induce low levels of neutralizing antibodies [54,91]. Genes encoding B and T cell epitopes delivered via plasmid also did not produce satisfactory results [92,93]. The precise explanation could not be found, but efforts were made to incorporate proteins stimulating the immune system, such as Bcl-xL anti-apoptotic and bovine IL-18 plasmid CDNA, in the vaccine formulation, which improved the results [94,95]. Similarly, an attempt was made to improve DNA vaccines with use of a purified recombinant FMDV-specific multi-epitope protein (rMEG990) and an optimized sindbis virus replicase-based DNA vaccine expressing this protein; slightly improved results in India were reported [96]. DNA vaccines hold promise for future vaccines similar to modified LAVs. More time is required before DNA vaccines can be considered for use in Indian settings.

### 2.7. Peptide Vaccines

This set of vaccines consists of immunogenic peptides either synthetic or expressed in eukaryotic or prokaryotic system [97]. As there is no handling of a live virus, the highly purified vaccine formulation with the desired proteins can be produced without the need for a biosaftey level III facility [98]. However, as with DNA vaccines, peptide vaccine offers incomplete protection and requires multiple boosters [99,100]. However, few reports describe full protection in pigs conferred by a peptide FMD vaccine [101,102]. Several methods of antigen delivery including transgenic plants or plants infected with recombinant viruses, etc., have been evaluated with partial success [98]. This vaccination strategy (among others) is being currently used in China [100].

Considering the involved cost/benefit ratio and considering the vast animal population of India to be covered, the use of peptides for preventive FMD control does not seem to be a viable alternative.

### 2.8. Plant Based Recombinant Vaccines

Large scale production of recombinant vaccines in plants has been shown as a promising biotechnological tool [103]. Theoretically, plant based vaccines offer several advantages, such as freedom from the need fora cold storage, biosafety level III facility, and diminished cost of production. Efforts were made to develop these vaccines in plants against various viral diseases, including FMD. The VP1 structural protein was expressed in alfalfa (*Medicago sativa*) and its potency was evaluatedin mice [104,105]. Similarly, improved expression of VP1 was shown in chloroplasts of tobacco (*Nicotiana tabacum*) [106]. However, the technology had several other challenges, including incomplete protection in large animals and pre-processing of leaves before feeding in some cases. These studies were limited in numbers and currently do not seem to be viable alternatives for the control program.

### 2.9. Potential Use of Immunomodulatory Molecules

Induction of earliest protection and long lasting immunity are the two parameters that still demand improvement. The available vaccine offers protection no less than 3–4 days and, similarly, the duration of protection lasts only for 4–6 months of vaccination. Improvement of adjuvants or addition of co-stimulatory molecules can significantly improve these two qualities of the vaccines, hence noteworthy work has been conducted to evaluate the immunomodulatory molecule for the improvement of immune response by the current FMD vaccines. In a study, it was demonstrated that the addition of immunopotentiator agent CVC1302 can significantly enhance the immune efficacy and the protective ability of the FMD vaccine in pigs in terms of long lasting antibodies [107]. Similarly, Poly(I:C) combined with multi-epitope protein vaccine completely protects against virulent FMDV challenge in pigs, which reduces animal to animal variations in both cellular and humoral immune response after vaccination with synthetic protein based vaccines [108]. Similar efforts have been made in India. In a study, guinea pigs immunized with VLP + CpG vaccine showed markedly higher cell mediated immunity (CMI) in comparison to the conventional vaccine group, as evident from higher levels of IgG2 than IgG1 [109]. In another study, liposome-based oil emulsion platform showed improved immunity in cattle [110]. After comparative analysis of several adjuvants, the Montanide ISA-201 adjuvanted FMD vaccine was found to better induce enhanced immune responses and protective efficacy in cattle [96].

The adjuvant effect of porcine interferon-alpha (PoIFN-alpha) was shown to be effective for enhancing the immune efficacy [111,112,113]. Experimental study also demonstrated enhanced immunogenicity of the conventional vaccine, even with the use of a suboptimal dose of FMD vaccine if co-administered with synthetic RNA transcript encoding for type-I interferon in pigs. Higher levels of anti-FMDV titers at late times post-vaccination and higher specific T-cell response and protection levels against FMDV challenge were also observed [113,114]. To further extend this work, recombinant adenoviruses for the simultaneous expression of porcine alpha and gamma interferons (Ad-porcine IFN-αγ) as well as threesmall interfering RNAs (Ad-3siRNA) were developed, and enhanced antiviral inhibitory effects were demonstrated [115]. The strategy was demonstrated as effective in pigs, which were challenged 1–2 days post vaccination [116].

Several other immunomodulatory molecules have also been investigated with the objective to achieve improved immune response. In one study, evidence of the activation of interferon regulatory factor 3 (IRF3)—a key transcriptional regulator of type I interferon (IFN)-dependent immune responses after transfection of swine and bovine cells with transcripts corresponding to the FMDV 3′UTR—was observed [117]. Baculovirus was evaluated in order to prolong the immune response. Inactivated vaccines co-inoculated with baculovirus showed early protection [118]. Interleukin-2 was found to potentiate foot-and-mouth disease vaccinal immune responses in mice [119]. Interleukin 15 has been known to improve the cell mediated immune response and is also involved in the maintenance of memory T and B cells. The role of bovine IL-15 as an adjuvant to inactivated FMD vaccine in a guinea pig model seems promising, showing improved levels of neutralizing antibodies for a period of six months [120]. However, the findings are yet to be evaluated in cattle and buffalo before being recommended for FMD control programs in India.

## 3. Epidemiology of FMD in India

**Figure 3:**
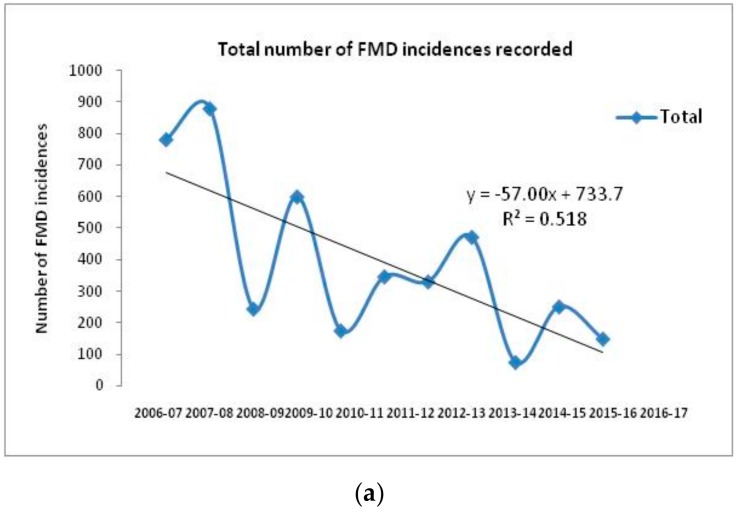

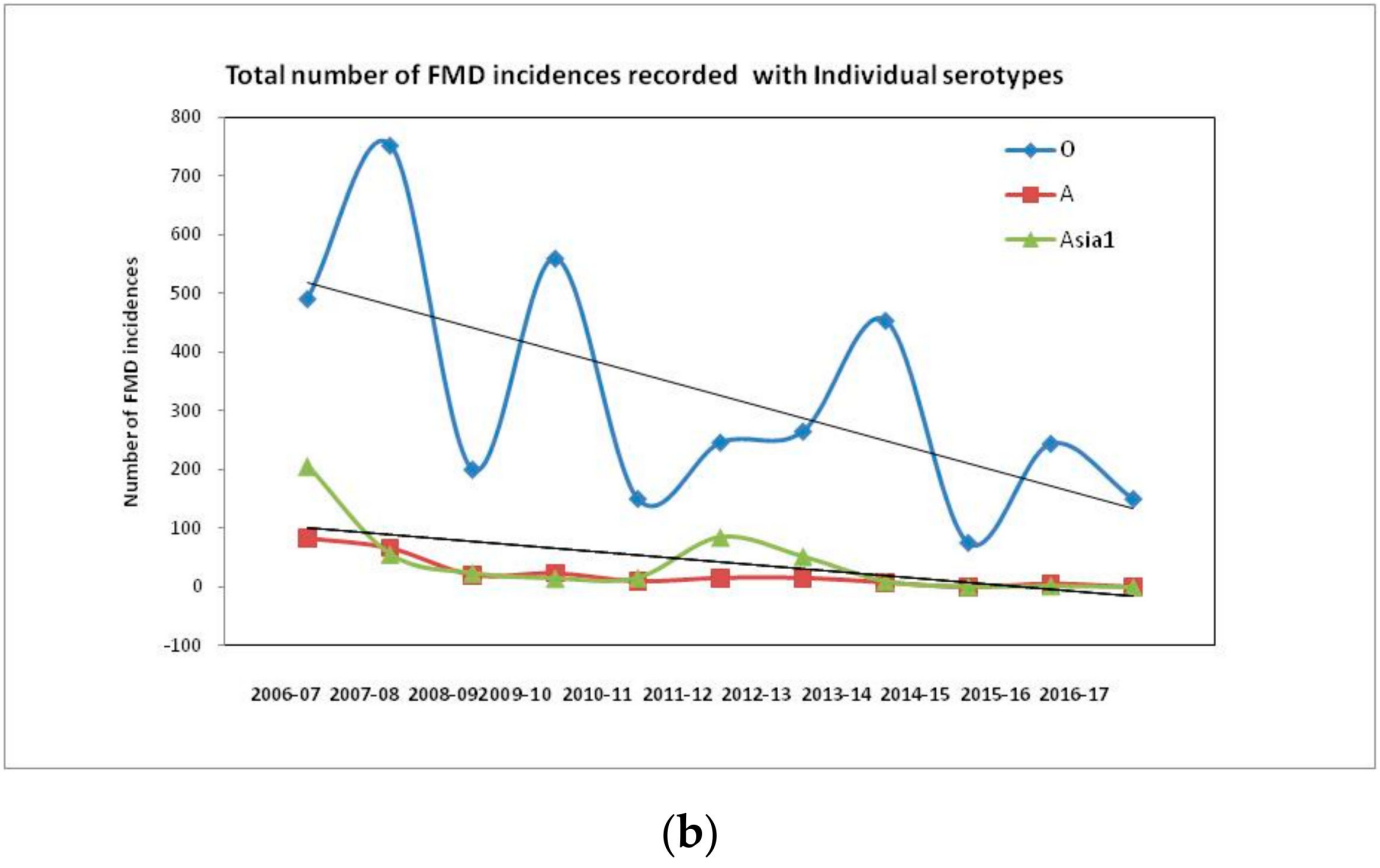
Total and serotype-wise temporal distribution of FMD outbreaks during the period from 2006–2017. The outbreak pattern was analyzed by simple linear regression with the coefficient of determination and the best fit line along with slope (R^2^) indicatinga gradual decrease in FMD outbreaks, which could be attributed to the efforts made in the FMD control program (FMDCP). (**a**) Total FMD outbreaks/year during 2006–2017, showing the consistent drop in number of FMD incidences. Outbreaks also appeared to fluctuate every alternate year, which can be attributed to the infection immunity. (**b**) Serotype O outbreaks that were in majority, showed similar observations as total FMD outbreaks. Serotype A outbreakssharply reduced after commencement of FMDCP. However, during the period of 2010–2011, there was slight surge in serotype A outbreaks due to VP3^59^-deletion group. Serotype Asia 1 outbreaks showed sudden reduction in incidences after implementation of FMDCP, except for the year 2011–2012 [29].

Currently, serotypes O, A, and Asia 1 are prevalent in India [29]. SAT serotypes have never been detected, whereas serotype C has not been detected in India since 1995 [26]. Historically, serotype O has dominated field outbreaks, followed by serotype Asia 1 and A (Figure 3, [26]). The temporal distribution of FMD cases with different serotypes over a period of 10 years in India indicates a gradual decrease in the number of FMD outbreaks associated with the progressive improvement in herd immunity due to continuous vaccination under FMDCP since 2007. The total incidence of FMD and individual serotype specific incidences from 2006 to 2017 indicate a decreasing trend with occasional spikes in the number of outbreaks at different time points (Figure 3). The undulating trend could be ascribed to the infection immunity, which determines the occurrence of an outbreak in subsequent years (Figure 3). Introduction of replacement stock could be one of the factors behind the resurrection of the disease. Although the proportion of FMD cases due to serotype O is very high when compared to thecases due to serotypes A and Asia 1, comparison of the serotype specific temporal distribution reveals that the incidence of serotypes A and Asia 1 increased only when FMD incidence due to serotype O was low, indicating the dominance of serotype O in the field outbreaks. Therefore, since 2003, a trivalent vaccine incorporating only three strains (O, A, and Asia 1 serotypes) has been implemented, as serotype C outbreaks have not been recorded since 1995. Since the implementation of the FMDCP, uniformity in virus strains has been regulated by the state agency, the Department of Animal Husbandry, Dairying, and Fisheries (Ministry of Agriculture and Farmers Welfare), and all vaccine manufacturers have used the same vaccine strains to produce inactivated vaccine for domestic use.

With the major expansion of the FMDCP covering 221 districts during 2009 and 2010, there have been markedly fewer FMD outbreaks, especially in the southern peninsular region of India, where all the districts were covered [26,28]. However, in 2013, there was a wide-scale outbreak of FMD [30] covering 631 villages during the period spanning January 2013 through February 2014. Of these, 472 outbreaks were confirmed by laboratory diagnoses, and the serotypes were characterized. All outbreaks in the southern peninsular region were reported to be due to FMDV serotype O belonging to the Ind2001 lineage [31]. Approximately 50% (228 of 472) of these outbreaks occurred in the four states of the southern peninsula, which have been covered under the FMDCP since 2009 [30].

### 3.1. Circulating Virus Pool

The circulating serotypes of FMDV in India are regularly monitored by nucleotide sequencing of capsid genes to determine the emergence and the re-emergence of virus variants. Detailed molecular phylogenetic analyses of individual serotypes have been studied, and the data have been used for selection of new vaccine candidates. Recently, an integrative phylogenetic analysis of prevailing lineages of the serotypes O, A, and Asia 1 in Southern Asia was presented [121]. In this section, we briefly describe the different lineages of these serotypes that have emerged and re-emerged in India to underline the importance of routine virus monitoring and vaccine matching.

### 3.2. Serotype O

Serotype O continues to dominate the field outbreaks in India with relatively low appearances of serotype A and Asia 1. FMDV serotype O Ind2001 lineage within the Middle East-South Asia (ME-SA) topotype is the major cause of recent FMD incidences in India. During the same time window, this lineage was reported from Libya. This lineage was responsible for most of the outbreaks during 2013 and 2014 in India and has been in circulation in India since 2008, along with the Pan Asia lineage [31,122]. The isolates of sub-lineage Ind2001d obtained during 2013 had few changes in the non-coding and the coding regions of the viral genome compared with those isolated from the previous year [31].

### 3.3. Serotype A

Serotype A virus is genetically and antigenically the most heterogeneous of all the FMD serotypes [123,124]. Since 2001, four genotypes of serotype A have been circulating in India [125]. A divergent and unique lineage that emerged has an amino acid (aa) deletion at position 59 of VP3 (and thus is termed the VP3^59^-deletion). This group has been predominant in field outbreaks since 2002–2003 [125]. This single amino acid deletion at an antigenically critical position in the structural protein VP3 is considered to be a major evolutionary jump and was most likely due to immune selection pressure. Since 2015–2016, all the prevailing serotype A clusters with genotype 18 have grouped only in the clade 18c of the VP3^59^-deletion lineage [125]. Interestingly, all the field outbreak strains during 2015–2016 harbored the VP3^59^ deletion, reflecting the emergence of exclusive dominance of the VP3^59^ deletion group.

### 3.4. Serotype Asia 1

Previous studies on 1D/VP1 gene-based phylogeny demarcated Indian serotype Asia 1 field isolates into three major lineages: B, C, and D. Lineage B includes the currently used serotype Asia 1 vaccine strain, IND 63/1972. This lineage was last recorded in the year 2000 [126]. The isolates of lineage D emerged late in 2001 and dominated between 2002 and 2004. Lineage C dominated the Asia 1 field outbreaks between 1998 and 2002, disappeared between 2001 and 2004, and re-emerged as the predominate lineage since 2005 [127]. Based on the serum neutralization data, no evidence of vaccination failure of serotype Asia 1 has been observed in the last two decades. However, Ullah et al. [128] inferred vaccination failure due to the emergence of heterogeneous serotype Asia 1 FMDV compared to the Indian vaccine strain (IND 63/1972) based on complete VP1 nucleotide sequencing data during 2012–2013.

## 4. FMD Monitoring in India

India has a huge livestock population with a susceptible population of 500 million (cattle, sheep, goat, pig, buffalo, yak, and mithun) apart from the wild life. Monitoring of the disease status in the population relies on the effective diagnosis and the rapid reporting system for disease epidemics. A network of laboratories participating in the All India Coordinated Research on FMD (AICRP on FMD) monitors FMD in real-time across the country. FMD outbreak diagnosis in the field and the reporting and vaccination program are monitored by the Animal Husbandry Department of each state government, with technical assistance provided by central government research institutes. A network of 23 laboratories has trained personnel for FMD sampling and diagnostic testing, serotyping, sero-surveillance, and sero-monitoring [26]. Data collected and laboratory data are shared on a real-time basis through an online laboratory information management system. These regional laboratories are situated in various parts of the country so as to act rapidly in the event of FMD outbreaks. A tool box with all the required assays for FMD diagnosis and monitoring designed as per local requirements was put in place and is used by the network of laboratories reviewed in [129]. Frequent refresher training programs are organized for these laboratories.

**Figure 4:**
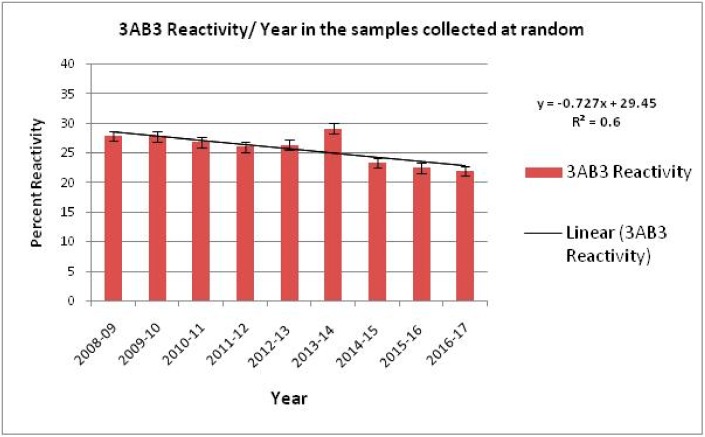
Proportion of animal population reacting with FMDV Non-structural proteins 3AB3 from 2008–2017, indicating a gradual decline in the reactivity that can be attributed to the FMDCP. However, an increase in animal population reacting to NSP was observed in 2013–2014, during which an FMD outbreak occurred. The best fit line along with the slope (R^2^) indicates a decrease in NSP reactors. Source: Annual Report DFMD 2008–2017.

Samples are submitted for detailed virological examination in the Directorate on Foot and Mouth Disease (DFMD) center, where antigenic and genetic characterizations of the field isolates are carried out. Routine sero-surveillance is carried out under the National Sero-Surveillance Program, which was launched in 2008 [29]. DIVA is accomplished by the detection of antibodies against 3AB3 non-structural proteins [33,130]. One hundred representative samples per district from cattle and buffalo are collected each year throughout India. These are analyzed by 3AB3 indirect ELISA. Gradual reduction in reactive non-structural proteins over a period of time has been observed (Figure 4). However, compared to the reduction in the number of FMD incidences during this period, sero-prevalence has not declined in the same proportion. This could be attributed to circulation of FMDV in vaccinated animals without the development of clinical signs.

**Figure 5:**
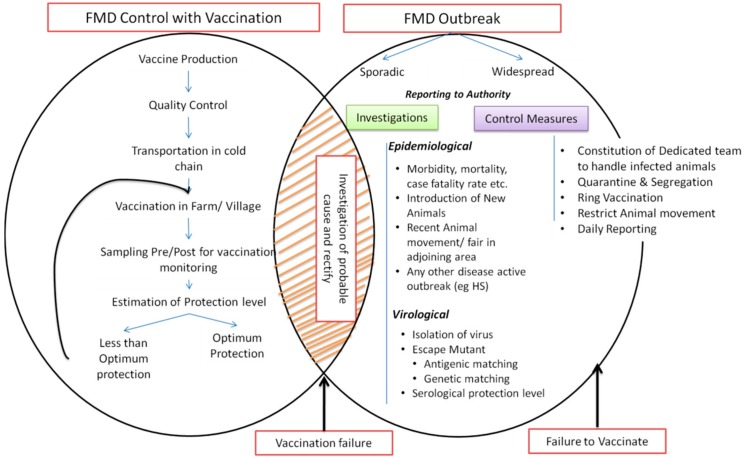
Schematic representation of vaccination based FMD control program in India. The figure describes the general procedure of vaccination and post vaccination monitoring and measures to be adopted for the investigation of FMD incidences.

Post-vaccination sero-monitoring is systematically conducted under a national sero-monitoring program [26,28]. Increases in the antibody titers 21–28 days following vaccination to O, A, and Asia 1 serotypes are measured by a quantitative ELISA. The details of the post-vaccination sero-monitoring were described previously [28]. Post-vaccination antibody titers indicate the level of protective immunity in the herd, which helps to refine the implementation of the control program. The schematic overview of the FMD vaccination and post-vaccination monitoring is presented in Figure 5.

## 5. Immunobiology of FMDV and Vaccine Failures: Challenges and Solutions

A drastic reduction in FMD outbreaks in terms of number of incidences and longevity of the outbreaks has been observed since the introduction of mass vaccination in India [29]. However, sporadic FMD events of variable intensity continue to occur despite vaccination. Generally, the incidence of FMD with variable intensity in vaccinated herds/areas is due to vaccination failure. The factors responsible have been classified as either failure to vaccinate or vaccination failure [34,131,132]. It is also possible that there is an interplay of more than one factor. The plausible factors are depicted in Figure 5.

### 5.1. Failure to Vaccinate

Failure to vaccinate is different from vaccination failure, where a large proportion of the population remains unvaccinated even after vaccination campaigns due to administrative/technical reasons. The most common factor for the occurrence of FMD in vaccinated areas in India is generally perceived as failure to vaccinate, which has been indicated by a low level of herd immunity in sero-monitoring studies. If animals are not vaccinated at all, or if only a few are vaccinated, there is high risk that the disease may flare up in adjacent areas. Failure to vaccinate can be due to a variety of reasons, including shortage of vaccine supply, improper transportation of vaccines (a break in the cold chain), lack of trained staff for vaccination, or anti-vaccine attitude by a few animal owners. Transient reduction of milk yield in the milch animals is a major deterrent for the owners to undertake vaccination, leading to poor vaccination coverage. To monitor low vaccination coverage, a systematic post-vaccination sero-monitoring is advocated, which can permit the identification of risk areas and resultsin corrective actions by the authorities.

### 5.2. Vaccination Failure

Vaccination failure describes clinical incidences of FMD in appropriately vaccinated animals. Although vaccination failure is a rare phenomenon, it is a more serious threat, as it may not be mitigated with management practices alone but also requires technical intervention. Identification of the precise reason for vaccination failure needs to be investigated to take corrective actions for the effectiveness of the control program. The plausible factors responsible for vaccination failure are discussed herein.

#### 5.2.1. Vaccine Strain Matching with Circulating Virus

To confer protective immunity, the vaccine strain should be antigenically related to the circulating viruses. Due to the high rate of virus mutation, strain differences can result in the failure of vaccination to protect against the diverging field strains [21,133]. The emergence of an antigenic divergent group within a serotype is common, owing to the error prone replication of the FMDV RNA genome in the presence of antibody selection pressure [123]. These antigenic divergent groups can be neutralized by vaccine induced antibodies with variable efficiency. Hence, regular vaccine matching is essential to evaluate the suitability of a vaccine candidate in providing protection to all the circulating strains. Vaccine matching in India is routinely performed using the Two-Dimensional Microneutralization Test (2D-MNT) [134] at FMD referral laboratories, including Directorate on Foot and Mouth Disease [122,123,124,125]. For this purpose, antigenic differentiation/vaccine matching of field isolates is important to understand the epidemiology of the disease and to select suitable vaccine strains. This provides important clues to confirm breaches in vaccinal immunity that are evident by the appearance of new antigenically divergent field strains, as well as to decide on the suitability of vaccine strain(s) to be used in a particular situation as effective prophylactic and control measures for the disease.

Until 2003, the O/TNN 24/1984 and the O/IND/R2/1975 strains of serotype O were incorporated in the vaccine formulation [122]. After 2003, it was observed that O/IND/R2/1975 provided protective immunity to all the circulating strains in the country. Hence, O/TNN24/1984 was discontinued from the vaccine formulation. Recent results of vaccine matching for serotype O have indicated that >90% of the serotype O isolates obtained from different parts of the country were antigenically related to O/IND/R2/1975 (*r* value >0.3) [29]. Emergence of an antigenic variant in an endemic country is not uncommon, and the currently used vaccine strain, O/IND/R2/1975, is still able to provide near-optimal antigenic coverage to the circulating field viruses. Similarly, strain IND 17/1977 of serotype A was replaced with strain IND40/2000 in 2009 upon the emergence of the antigenically diverse serotype A virus lineage [123,124]. However, considerable antigenic drift of the recent serotype A field viruses from the Indian vaccine strain prompted a search for an alternate vaccine candidate [123]. In serotype Asia 1, IND 63/1972 strain provides adequate antigenic coverage to all the circulating strains of serotype Asia 1 [127]. All the field isolates demonstrated an *r* value of >0.3 using the bovine vaccinal serum against IND63/1972 strain. Hence, these strains are still being used in vaccine formulations [29].

#### 5.2.2. Vaccine Quality

The quality of vaccine formulations is crucial to the success of the vaccination-based FMDCP. Potency of vaccines is assessed by the Pharmacopeia and OIE manual guidelines. Vaccine potency is defined by the OIE as the “concentration of the immunologically active component” [34]. Potency according to this definition is often measured by vaccine manufacturers through the quantification of an antigen, thus a dose of a vaccine delivers a known antigen “payload” [135]. The conventional method of evaluating the effectiveness of FMD vaccines is by experimentally challenging vaccinated and unvaccinated control animals. Although inconsistent with the OIE definition of potency, these evaluations are commonly known as “potency tests”. The first of these tests estimates the 50% protective dose (PD_50_) value, which is recommended in European Pharmacopeia. The PD_50_ value is defined as the dose that protects 50% of those under the particular challenge regimen [135]. Standard vaccine has a potency of minimum 3PD_50_, whereas as a high payload vaccine contains a minimum of 6PD_50_. In India, generally a vaccine with a potency of 3PD_50_ or more is used [26]. The current inactivated vaccine formulation is thermolabile and requires cold chain storage of 2–8 °C until it is administered into the animals [135,136]. Break in the cold chain results in loss of antigenic mass (146S virus particle) in the vaccine, which adversely affects vaccinal immunity. However, vaccination with poor quality vaccines could be detected by post-vaccination sero-monitoring.

#### 5.2.3. Impaired Immune Response to Vaccine

Variations in the immune response to inactivated FMD vaccines have been observed within and between animal species, which could be associated with several factors. Different species generate antibody diversity in fundamentally different ways, which makes it difficult to develop a suitable laboratory model for the examination of potency [42]. Although antibody secreting cells present in the lymphoid tissue of the respiratory tract produce antibodies against FMDV upon aerosol infection as early as four days post-infection, viruses are secreted from the infected animals before the appearance of clinical symptoms [137]. Initial T cell independent clearance of the virus occurs through IgM antibodies, which are abundantly present locally as well as systemically. It has been demonstrated that the inactivated vaccines also prime the B cells, which extend to the lymph nodes draining the respiratory system and respond rapidly upon aerosol infection of FMDV [138].

Potential roles of non-neutralizing opsonic antibodies have also been demonstrated. These antibodies facilitate the uptake of bound FMDV by dendritic cells, which are potent immune modulators [139,140]. These findings suggest the need to consider immune complexes in vivo in target species during infection and vaccination and to explore how this new knowledge can be exploited to improve immunity [140].

The role of the T cell dependent antibody response is still unclear during the early response to FMDV infection [141]. The little evidence that is available indicates that bovine gamma-delta T cells are the regulatory equivalent of murine and human Foxp3-positive T regs and also demonstrates the ability of these cells to significantly impede FMDV-specific CD4 and CD8 T cell proliferation in vitro [142,143]. Further studies are required to confirm these indications in different breeds of Indian cattle and buffalo, as breed susceptibility to FMDV varies amongst different breeds of animals. Studies are also required for the complete understanding of other important immune responses such asthe CD8 T cell response, thus vaccines can be designed for better induction of the immune response [144].

#### 5.2.4. Break in the Herd Immunity

Herd immunity offers indirect protection from infectious disease that occurs when a large percentage of a population has become immune to an infection, thereby providing a measure of protection for individual animals that are not immune. In endemic settings, herd immunity is vital in FMD epidemiology and determines the outcome of outbreak in terms of number of animals affected and longevity of the outbreak [18,145]. The susceptible animal population must be re-vaccinated every 4–6 months owing to the short-lived humoral immunity [145,146,147]. Experiences gained from Europe and South America suggest that systematic vaccination can successfully eradicate FMD, provided the level of herd immunity is maintained [148,149]. Quantifying and deploying effective vaccination coverage at a population level is an essential component of any FMDCP in an endemic setting [150]. Uncertainty in vaccination coverage estimates could be addressed through improved record keeping, including the use of vaccination record cards, as recommended in the FAO-OIE Post-Vaccination Monitoring Guidelines. Post-vaccination sero-monitoring helps in the identification of areas with a low level of herd immunity. As mentioned earlier, there was a sudden increase in the number of FMD cases in India in 2014. The analysis of the sero-monitoring data of that period revealed a strong correlation between the incidence of FMD and areas not covered under the vaccination program [28].

Vaccine efficacy and vaccination coverage are the two important factors in generating the desired level of herd immunity against FMD in the field [151]. It is even more important to consistently maintain the herd immunity. Any break in herd immunity provides a window of opportunity for the silently circulating virus to spread to the entire population [34]. A small proportion of the targeted animal population always remain unvaccinated, as 100% coverage during the vaccination campaign is impractical [136,147]. For example, calves <4 months of age and pregnant animals in their third trimester are left unvaccinated during a vaccination drive. In addition, some animals in villages or herdsare debilitated or chronically sick and are not be vaccinated. However, vaccination-induced herd immunity is arguably more important over individual animal protection in FMD endemic regions. Although successful vaccination programs generating herd immunity prevent major disease outbreaks, establishment of herd immunity is made more difficult due to varied host responses to vaccination, wide circulation of the virus in the presence of a large number of a susceptible population, and vaccines that provide protection only for a limited period of time [32]. Apart from providing direct benefits to the vaccinated animal, herd immunity also confers an indirect benefit to the unvaccinated animals by reducing the virus transmission rate to near-zero [152]. Complete protection in the herd is difficult, if not impossible, to obtain. However, it was estimated that herd immunity of 80% or more using a vaccine strain matching the field strains would result in protection from clinical FMD outbreak [28,147]. The vaccinated animals reduce the basic reproduction number to a negligible level, from which the disease can spread in the population. Literature shows that the immunity threshold, 1–1/R0, where R0 is the basic reproduction number (number of susceptible animals that become infected compared to infected animals), can be used as a yardstick for immunization coverage that can control the target infections [152,153,154]. The incidence of an infection will decline if the proportion of immune animals exceeds the herd immunity threshold. However, because of the considerably longer carrier status of FMD (>8 months) and the low duration of protection offered by a vaccine, decline in the herd immunity may lead to the spread of the virus infection [18].

#### 5.2.5. Duration of Protective Immunity

Protection from a primary course of vaccination typically lasts for approximately 4–6 months, depending on the potency of the vaccine [20,135,140]. Hence, animals are re-vaccinated depending upon the epidemiological situation in the country [20,146]. Based on the quantification of the antibody titers, the antibody decay is estimated [28,32]. In India, it has been demonstrated that a rapid antibody decay in vaccinated animals, especially against serotype O, can cause a break in the herd immunity with a window of infection at 5–6 months post-vaccination. This creates the opportunity for spread of the virus in the population. Efforts are being made to improve the duration of immune protection. To reduce the risk of break in the herd immunity, the vaccination policy should be framed considering the local epidemiological factors and other risk factors, such as frequency of animal mixing. Knight-Jones et al. [155] suggested using a vaccine of higher potency and/or a higher number of vaccine inoculations to decrease these gaps in immunity, particularly in younger cows. In 2013 and 2014, very large numbers of FMD cases were observed [30]. Examination of the association of herd immunity with outbreaks in 2014 revealed that the incidence of FMD began to rise at five months post-vaccination, when vaccination-induced immunity began to wane [28].

#### 5.2.6. Maternally Derived Antibody Inhibition

Vaccination failure in neonates has been studied less than in adults. Calves are generally not vaccinated during a vaccination drive due to the chance of vaccination failure [150]. The presence of FMD susceptible calves in a herd increases the risk to vaccinated adults. Maternally derived antibodies hinder an effective immune stimulation by inactivated vaccines. Thus, it is possible that neonates remain susceptible to FMDV even after vaccination. The neonatal immune system of the FMDV target species is still far less characterized in comparison to the adults [42]. Anti-FMDV antibodies present in the colostrum may prevent the development of the anti-FMDV IgM response and could interfere with the production of virus-neutralizing antibodies after vaccination in the young calves [156,157]. Recent research has focused primarily on determining the suitable dose, the booster, and the timing of FMD vaccination in young calves. Little research has focused on determining the T cell response in the young calves after vaccination. Some suggestions have been provided for inducing neutralizing antibodies in calves with maternal antibodies. They include use of oil-based adjuvants instead of aluminum hydroxide-based adjuvants [158], an intratypic heterologous vaccine that is somewhat antigenically different from the vaccine given to dams [42], and the use of adeno-vectored vaccines, as was successfully demonstrated for swine influenza vaccines [159]. However, the overall findings are inconsistent and highlight the issue of potential differences in efficacy of vaccines in young animals. Work to date suggests that neonatal calves are able to mount responses to commercially available FMDV vaccines but only in the absence of high levels of maternal antibody and perhaps only with the “right” adjuvant. Further study of neonatal immunity with particular reference to the T cell compartment in conjunction with side-by-side comparisons of responses to different adjuvants is required.

#### 5.2.7. FMDV Persistence in Recovered Animals

FMDV carriers have been defined as animals that shed infectious virus in the oropharyngeal fluid later than 28 days after infection [160]. Carrier ruminant animals can only be identified by detection of an FMDV antigen or a genome in oro-nasopharyngeal fluids in endemic settings [8]. Detection of antibodies against structural and non-structural proteins of FMDV makes little sense in endemic settings with vaccination, as there is no difference in the serological profiles of the infected and the carrier animals [161,162].

FMD carrier animals secrete the virus for more than one year following recovery from natural infection [163]. These animals carry the virus in the oropharynx but do not exhibit symptoms. The epithelia and the lymphoid germinal centers of the oropharynx have been identified as sites for FMDV persistence [164]. However, the exact site and the mechanism of virus persistence are still unknown [7]. In some clinically and sub-clinically infected ruminants, FMDV can be isolated from oropharyngeal fluids and/or tissues >28 days after infection [163]. This condition is referred to as persistent FMDV infection, and such animals are referred to as “carriers” [3,165,166]. Recently, FMD transmission via the persistent transfer of oropharyngeal fluid from infected to naïve animals was demonstrated [6], indicating that the presence of carrier animals in a herd can have profound implications for international and domestic trade [167]. It has been proposed that new virus variants are produced in buffalo during the prolonged carriage after acute infection, which may spread to cause disease in livestock populations [164]. Even in the vaccinated farm, a small proportion of animals may remain as carriers without clinical symptoms but capable of secreting FMDV into the environment. These animals have been identified as potential risks for the spread of FMD outbreaks if mixed with naïve animals or if there is a break in the herd immunity.

#### 5.2.8. Other Factors

In addition to the factors mentioned above, several other factors may have a role in vaccination failure.

##### Virus Circulation in Other Ruminant Species

Although the risk of transmission of FMDV from one species to the other is not well documented, there is an unarguable threat posed by these animals to the herd [168]. Currently, only cattle and buffalo are being vaccinated under the FMDCP in India. Other livestock species, especially sheep and goats, are randomly vaccinated. These species are covered under regular vaccination programs only in some areas. Similarly, swine species, which, after infection, amplify the virus to a very large extent, are also not covered under the FMDCP. Hence, cattle and buffalo in those areas have a potential risk of coming down with the disease. Epidemiological data are still scant, and further studies are needed.

##### Animal Stressors

The role of stress in animals on the break in immunity also has not been documented extensively. However, environmental stress certainly is crucial in reducing the immunity and heightening the susceptibility for infection. Animals experience stress mostly due to transportation and handling/restraint, extreme weather, inadequate nutrition, and certain physiological conditions such as pregnancy. Natural disasters, such as drought and famine, are also factors that indirectly influence the susceptibility. Inadequate food supplies compromises the immune status of animals. Moreover, the risks associated with import of food supplies also may heighten the risks of disease. FMD was reported for a prolonged period in Andaman and Nicobar Islands in 2005 after a tsunami [169]. It was unclear whether the animals already had the virus that subsequently flared up upon the environmental stress or whether the virus originated in the materials used to package the relief supplies. FMD has also been reported from many shelter farms established specifically to cope with natural disasters such as floods or droughts. The mixing of animals from different sources in these farms plays a crucial role in spreading disease, and stress certainly plays a role in increasing the susceptibility of the vaccinated animals to the disease. Further data need to be collected and analyzed to help in designing contingency plans. In addition, the role of other stresses, such as parasitic load, and the epidemiology of other infectious diseases also need to be explored to clarify the factors behind the break in the immunity.

##### Amount of FMDV Exposure to Vaccinated Animals

Some recent studies have highlighted the potentially crucial role of the risk of duration and the amount of virus exposure to a vaccinated animal in vaccination failure. All animals exposed to FMDV will not develop clinical symptoms. The amount of virus secreted from the infected animals determines the intensity of transmission of FMD in susceptible animals. Both clinically and non-clinically infected animals secrete the virus in the environment and act as a source of contamination for other animals. Virus secretion from recovered carrier animals that appear healthy is most threatening to the susceptible livestock animals. It has been estimated that clinically infected and recovered carrier animals secrete the virus in saliva and in nasal fluid for more than one year [163]. The amount of virus shed in the environment has also been quantified. In one study, the median peak level of virus shed in saliva was estimated to be 10^6.9^ copies/mL in unvaccinated carriers, 10^3.7^ copies/mL in vaccinated carriers, 10^6.5^ copies/mL in unvaccinated non-carriers, and 10^3.7^ copies/mL in vaccinated non-carriers [170]. The peak period of shedding occurred between two and seven days post-infection, irrespective of vaccination or carrier status [170]. The median total quantity of virus shed was 10^7.4^ copies/mL in unvaccinated carriers, 10^4.2^ copies/mL in vaccinated carriers, 10^7.0^ copies/mL in unvaccinated non-carriers, and 10^4.2^ copies/mL in vaccinated non-carriers. The median duration of shedding in saliva was 10 days in unvaccinated carriers, 2.3 days in vaccinated carriers, 10 days in unvaccinated non-carriers, and 2.5 days in vaccinated non-carriers. Similar studies from other species, especially pigs, are available [171].

A dry filter air sampling system developed recently [172] can be used to estimate the aerosol contamination of FMDV present as a virus plume, which can be an associated risk factor. Application of a transmission networking model provides valuable insights in the understanding of disease transmission and the identification of farms or areas at risk of FMD spread in real-time and retrospective analyses. The information will be useful in tracing the spread of disease in order to establish effective prevention and control strategies for future FMD outbreaks and to effectively define the risk associated with the quantity of FMDV exposure.

## 6. Tackling the Challenges

FMD endemicity in India has serious consequences for Indian livestock farmers whose livelihoods depend on their animals. Also, huge direct and indirect losses due to FMD are a major concern to the national economy. Therefore, it is imperative to address the challenges involved in FMD control.

Effective livestock management practices, including proper vaccination and restricting the movement of animals from risk prone areas, could help manage the disease risk. To help achieve the goal of disease-free zones in the country, it is essential to have cooperative strategies involving central and state governments and the livestock farming community. Preventive vaccination has been successful in many South American countriesin not only controlling the disease but also in eradicating it. Therefore, improving vaccines needs to be livestock research priority. Also, considering that early use of a highly potent vaccine during early notice of an outbreak could reduce virus shedding and restrict the disease transmission, this strategy needs to be brought into practice.

Along with the vaccination program controlling FMD, it is vital that field diagnostics be genuinely used. An emphasis on early diagnosis together with blocking disease transmission will aid in effective disease containment. It is essential to develop and evaluate simple pen-side diagnostic tests to detect the disease in the field.

Additionally, improved methods are needed for the rapid selection of vaccine strains to match the circulating field strain, and the selected vaccine must be available in sufficient quantities. This will result in an effective regional vaccination strategy.

Furthermore, effectiveness of the FMD vaccine under field conditions may vary from the vaccine potency resulting from testing under experimental conditions. Optimal vaccination techniques must be established by trained vaccinators. Also, treatment for other prevailing immunosuppressing diseases must be carried out promptly by field veterinarians.

Effective biosecurity measures have to be in place to control the transmission of FMD between herds. In a country such as India, where culling of infected animals cannot be practiced, it is advisable to identify and quarantine clinically diseased and suspected animals so as to reduce virus transmission and allow animals to recover. The quarantine practice must be followed compulsorily when introducing newly purchased livestock onto the farm.

## 7. Conclusions and Future Prospects

Efforts need to be intensified to control and eradicate FMD from India. Success stories are available with respect to control and the eventual eradication of FMD in South American and European countries. Successful control and eradication of the disease in these countries using inactivated vaccines is well documented and available for other countries to follow the progressive control of the disease as envisaged in the PCP pathway. FMDCP was initiated in India in the year 2003. With consistent efforts, FMD has been controlled to an extent. However, sporadic outbreaks have continued to be reported from vaccinated areas. It was realized that the policies for control of FMD adopted in European countries need to be modified to suit the conditions in India before they can be implemented. FMD epidemiology is complicated in India because of its huge ruminant population. Socio-economic issues further complicate the implementation of a control program. In these circumstances, vaccination failure has severely reduced confidence in mass vaccination-based control programs. Failure to vaccinate is identified as the primary reason for most of the outbreaks. This factor will only be addressed by improving the veterinary infrastructure, including cold chain maintenance for vaccine transportation, and by training personnel for proper vaccination, strengthening real-time FMD monitoring including post-vaccination sero-monitoring, and related issues. In addition, an adequate supply of quality vaccine should be ensured at every phase of vaccination to avoid break in herd immunity. Efforts have already been made to develop new-generation vaccines that are economical, offer longer duration of protection, and are sufficiently stable to transport in the tropical conditions of India. These efforts need to be increased, including the use of animal models to replace inactivated vaccine formulation.

Until the time when vaccines can be refined, the plausibility of vaccinating animals three times annually could be explored to maintain the high titer of antibodies in the herd. A system for real-time surveillance and monitoring of FMD is in place. However, frequent training using the latest techniques needs to take place. The national government recently decided that the entire cost of vaccination will be paid for. Hence, it is anticipated that further improvement in FMD control should be realized soon, and that vaccination failure will be effectively addressed to increase the herd immunity in the population.
